# Supplementary material for: Genome-Wide Association Study of Early Vigour-Related Traits for a Rice (Oryza sativa L.) japonica Diversity Set Grown in Aerobic Conditions
Source: Biology (Basel). 2024 Apr 15;13(4):261. doi: 10.3390/biology13040261 (PMC11048181; doi:10.3390/biology13040261)
Supplement: Supplementary file 1 [file biology-13-00261-s001.zip › biology-2938273-supplementary.pdf]

Supplementary material

Table S1 The correlation matrix between mesocotyl length (ML), early vigour score (EVS), plant height (PH), days to emergence (DTE), biomass per plant (Biomass PP), biomass per m^{-2} and light interception (LI) in two glasshouse experiments (GH18 and GH21) and three field experiments (FIELD19, FIELD20 and FIELD22)

	ML GH18	PH GH18	DTE GH18	Biomass PP GH18	PH GH21	DTE GH21	EVS FIELD19	LI FIELD20	PH FIELD20	DTE FIELD20	Biomass m^{-2} FIELD20	EVS FIELD20
PH GH18	0.68**											
DTE GH18	-0.36**	-0.53**										
Biomass PP GH18	0.59**	0.90**	-0.50**									
PH GH21	0.33**	0.42**	-0.16**	0.34**								
DTE GH21	-0.22**	-0.15*	0.12*	-0.11ns	-0.24**							
EVS FIELD19	-0.35**	-0.44**	0.25**	-0.42**	-0.44**	0.14*						
LI FIELD20	0.33**	0.44**	-0.22**	0.30**	0.37**	-0.12ns	-0.43**					
PH FIELD20	0.38**	0.52**	-0.24**	0.38**	0.48**	-0.10ns	-0.44**	0.64**				
DTE FIELD20	-0.34**	-0.37**	0.12*	-0.29**	-0.27**	0.20**	0.28**	-0.44**	-0.29**			
Biomass m^{-2} FIELD20	0.31**	0.40**	-0.22**	0.33**	0.24**	-0.03ns	-0.27**	0.53**	0.46**	-0.36**		
EVS FIELD20	-0.37**	-0.47**	0.24**	-0.37**	-0.37**	0.13*	0.45**	-0.66**	-0.72**	0.51**	-0.57**	
EVS FIELD22	-0.38**	-0.52**	0.31**	-0.46**	-0.49**	0.09ns	0.45**	-0.53**	-0.63**	0.25*	-0.40**	0.54**

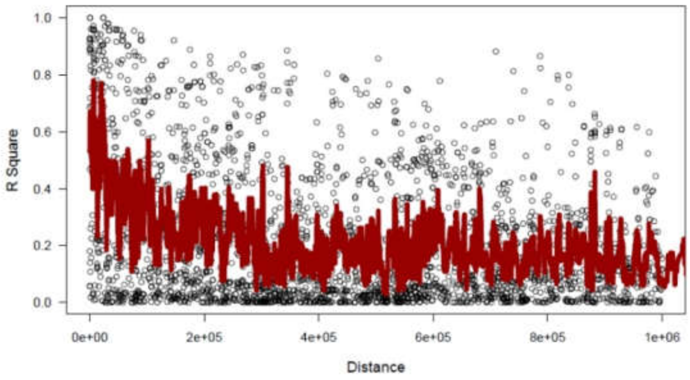

Figure S1 Linkage disequilibrium (R square) decay of marker-pairs over all chromosomes for the population
